# Illness perception about hepatitis C virus infection: a cross-sectional study from Khyber Pakhtunkhwa Pakistan

**DOI:** 10.1186/s12879-022-07055-5

**Published:** 2022-01-21

**Authors:** Shahan Ullah, Salamat Ali, Muhammad Daud, Vibhu Paudyal, Kawsar Hayat, Syed Muhammad Hamid, Tofeeq Ur-rehman

**Affiliations:** 1 Department of Pharmacy, Quaid-I-Azam University, Islamabad, Pakistan; 2 Department of Gastroenterology, District Headquarter Hospital Charsadda, Khyber Pakhtunkhwa, Pakistan; 3 School of Pharmacy, University of Birmingham, Birmingham, UK; 4 Department of Gastroenterology and Hepatology, Lady Reading Hospital Peshawar, Khyber Pakhtunkhwa, Pakistan; 5 Department of Statistics, Quaid-I-Azam University, Islamabad, Pakistan

**Keywords:** Brief Illness Perception Questionnaire, Hepatitis C, Khyber Pakhtunkhwa, Perception, Pakistan, Cross-sectional study

## Abstract

**Background:**

Hepatitis C virus (HCV) infection is a debilitating chronic health problem and can be fatal if left untreated. Illness perceptions are self-manifested beliefs that influence the ability of individuals to cope with their disease and perceive it as manageable or threatening condition. Limited evidence is available from low resource settings regarding patient perception about HCV. In this study, we aimed to assess the perception of individuals with HCV, the impact of their sociodemographic and clinical characteristics on their HCV perception, and its link to patient-oriented treatment outcomes.

**Methods:**

A cross-sectional survey was undertaken enrolling individuals with HCV who attended Hepatitis C clinics at two hospitals of Khyber Pakhtunkhwa, Pakistan. Illness perception was measured using Brief Illness Perception Questionnaire (BIPQ). Descriptive statistics, Kruskal Wallis tests and Mann Whitney U tests were performed to study patient sociodemographic and clinical characteristics and to analyze the questionnaire results. Multivariable linear regression was used to assess determinants associated with perception scores.

**Results:**

Participants represented poor HCV perception and their overall mean BIPQ score was 43.35, SD = 13.15. Participants had a low degree of understanding about their illness (mean coherence score = 2.92, SD = 1.85). Individuals with more than four years, compared to less than one year, of estimated HCV infection were more likely to view that their illness would continue (mean timeline score = 6.27, SD = 2.50 versus 5.36, SD = 2.53; respectively, p < 0.01). Similarly, individuals with hepatic cirrhosis, compared to without, were more likely to attribute symptoms to their disease (mean identity score = 5.48, SD = 2.14 versus 4.89, SD = 2.38; respectively, p = 0.04). Female participants reported higher degrees at which the illness affected them emotionally (i.e., emotional representation) and lower coherence about HCV than males (p = 0.04 and 0.006, respectively). Individuals who did not achieve sustained virological response 24 weeks after treatment with interferon-based therapy, compared to treatment naïve individuals, reported lower trust in being successfully treated with newer anti-HCV agents (i.e., direct acting antivirals) (p = 0.029). However, multivariable linear regression revealed that no sociodemographic or clinical determinants were associated with a higher BIPQ score (i.e., more threatening, or negative perceptions).

**Conclusion:**

Individuals with HCV in Pakistan generally report threatening or negative views about HCV infection. Lack of trust in treatment efficacy was also apparent, especially in those who experienced failed anti-HCV treatments in the past. Healthcare professionals should consider these perceptions when treating individuals with HCV to optimize their compliance by aligning their perception with the high effectiveness of current anti-HCV therapies.

**Supplementary Information:**

The online version contains supplementary material available at 10.1186/s12879-022-07055-5.

## Background

Hepatitis C virus (HCV) is a significant cause of chronic liver diseases worldwide [1]. Hepatitis C is a blood-borne infection that causes acute and chronic hepatitis ranging from mild illness of a few weeks to life-long illness. According to World Health Organization (WHO), 71 million people are infected with this virus across the world. WHO estimated that in 2016 approximately 399,000 people died from HCV-related complications, mostly from hepatocellular carcinoma and liver cirrhosis [2]. Pakistan ranks second amongst the highest HCV prevalent countries globally, with a prevalence of 4.7% [3–5].

The concept of illness perception has gained importance in recent years. It is amongst the essential psychological variables accountable for assessing patient behavior in chronic diseases [6, 7]. Illness perception is based on Self-regulatory model of Illness (SRM) presented by Leventhal et al. in 1980, which is also described as the “common-sense model of illness representation”. This model represents a framework describing how individuals make sense of their symptoms and experiences during a health threat or diagnosis and how they follow certain coping behaviours subsequently [8].

Various studies have revealed that illness perception impacts coping behavior of individuals which is linked to their health-related outcomes [9–11]. Coping behaviours or strategies are behavioral and cognitive strategies adopted by individuals to manage stress associated with having to live with the disease (e.g., adapting certain kind of physical activity or actively diverting attention from the illness) [12]. Perceived lack of formal and informal social support, perceived lack of access to health services, stigmatization, presence of anxiety and depression, and fear of disease progression are some of the perceptions that impact coping behaviour of individuals with chronic illnesses [13]. Moreover, illness perception and health-seeking behaviour is also influenced by sociodemographic characteristics of patients such as age, gender, and employment status [14, 15]. Most of the individuals with HCV are unaware of their disease status, which might be due to lack of diagnostic facilities or lack of disease-specific counseling to promote behavioral changes that might reduce their disease progression or avert infection transmission [16].

Addressing patient perception at an early stage by healthcare professionals provides an opportunity to improve patient beliefs which can contribute positively to a wide range of patient-oriented treatment outcomes [17]. However, health care providers are usually unaware of the self-manifested perceptions of patients and are unable to educate and counsel them properly [18–20]. Modifying illness perceptions and engaging patients in self-care by healthcare professionals has been proven beneficial in a cohort of individuals with irritable bowel syndrome [21] and end-stage renal disease patients [22]. Similarly, studies have demonstrated the impact of SRM models in improving perception through engaging the patients in self-care programs in various chronic illnesses such as hypertension [23], Addison’s disease [24], asthma [25], human immunodeficiency virus [26], rheumatoid arthritis [27], epilepsy [28], cancer [29], and chronic obstructive lung disease [30]. In HCV infection, sustained virologic response (SVR) is considered a successful treatment result, which is the absence of detectable HCV RNA on blood testing after 12 weeks of completion of the antiviral therapy, and little focus is given to the role of perception in optimizing therapeutic outcomes. Literature shows that few studies have reported the impact of illness perception in HCV related treatment outcomes. Langston et al. [10] reported that positive illness perception has successfully improved the mental health condition and lowered the substance abuse in individuals with HCV. On the contrary, Wang et al. [11] and Nergiz et al. [31] associated poor HCV perception of individuals with their negative emotions and anxiety.

After low success rates of IFN based anti-HCV regimens in Pakistan, direct acting antivirals (DAAs) were introduced. DAAs have more than 95% SVR rates and are available in Pakistan [32]. Despite this evidence, there is often a misconception that HCV is an untreatable disease [33]. Whilst this could link to perceived urgency of treatment needs and adapting precautionary lifestyle by the general population, infection prevalence remains high when compared with developed countries [34]. Therefore, this study was aimed to explore the perception of individuals about HCV infection, the impact of their sociodemographic and clinical characteristics on HCV perception, and its link to treatment-oriented outcomes.

## Methods

### Study design, settings, and participants

A cross-sectional study was undertaken to recruit participants from two hospitals of Khyber Pakhtunkhwa (KPK): a tertiary care hospital from District Peshawar and a secondary care hospital from District Charsadda. Individuals with HCV (tested positive through serum HCV RNA PCR analysis), age ≥ 18 years, without any significant comorbidities such as liver cancer, renal failure, diabetes, cardiac problems, hypertension, and who gave informed consent were included in the study.

A questionnaire-based, researcher-administered survey was conducted between January 2018 and December 2018. A convenience sample of participants visiting outpatient Hepatitis C clinics of each hospital were included in the study.

### Data sources, variables, and measurement

A paper-based structured questionnaire was used for data collection. This questionnaire had two sections, one for information pertaining to sociodemographic and clinical history of participants and a second part consisted of questions on perception. Perception was measured through the “Brief Illness Perception Questionnaire (BIPQ)” [35]. Sociodemographic characteristics of participants like age, sex, qualification, occupation, and monthly income were gathered during interview. These characteristics not only provide background information about the participants but also influence the development of a specific perception in them. Clinical data like duration of illness (time spent after the participants’ blood tests showed positive results for hepatitis C infection through HCV RNA PCR test) and liver cirrhosis (from diagnostic ultrasound reports) were also obtained from the participants. BIPQ was translated into Pashto language according to WHO guidelines. BIPQ has nine items; five of these items represent negative perception outcomes (higher the score, higher the threatening view about illness) and include questions about consequences (“*How much does your illness affect your life?”*), timeline (“*How long do you think your illness will continue?”*), identity (“*How much do you experience symptoms from your illness?”*), concern (“*How concerned are you about your illness?”*), and emotional representation (“*How much does your illness affect you emotionally? e.g., does it make you angry, scared, upset or depressed?”*). Three items represent positive illness-related perceptions (higher the score, more benign or optimistic view about the illness) and include personal control (*How much control do you feel you have over your illness?*), treatment control (“*How much do you think your treatment can help your illness?”*), and coherence (“*How well do you feel you understand your illness?”*). The ninth item is an open-ended question that asks about the three most important factors responsible for HCV infection according to the participant beliefs (*“Please list in rank-order the three most important factors that you believe caused your Illness”*).

Each of eight items was scored on a Likert scale of 0 to 10. A higher item score represented a stronger endorsement of that item. Moreover, an overall score (Total score = 80) was computed by reversing the scores of personal control, treatment control, and coherence, and summing it with the scores of consequences, timeline, identity, concern, and emotional representation. A higher total score represented threatening or negative perception, and lower score represented a benign or positive perception about the infection [35].

### Study size and addressing potential of bias

All participants who visited outpatient clinics during study period were approached for the study. Interviews were conducted in a dedicated area of each hospital. Participants were briefed about the research purpose, and they were assured of their data confidentiality. Informed consent was taken before they participated in the study. Physicians and other healthcare professionals were not allowed to access the research data; however, they were briefed about the study purpose.

### Statistical analysis

Descriptive statistics were used to describe sociodemographic and clinical characteristics of study participants and scoring of BIPQ. Cronbach’s alpha was calculated to measure the reliability of Pashto version of BIPQ. Kolmogorov–Smirnov test was used for determining normality of data. Mann Whitney U test and Kruskal–Wallis tests were used to compare the study variables. Multivariable linear regression was performed to assess determinants associated with poor or threatening perception of participants based on BIPQ overall score. Overall score was taken as dependent variable adjusted for sociodemographic and clinical characteristics of participants (independent variables) like age, sex, qualification, occupation, duration of illness, presence of cirrhosis, and failed IFN based treatment in the past. Results with *p* ≤ 0.05 were considered statistically significant. SPSS V.21.0 and STATA 14.2 were used for data analysis.

## Results

### Sociodemographic and clinical characteristics of the participants

A total of 550 individuals fulfilling the inclusion criteria were approached for the study, and 525 provided consent for participation (95.4% response rate). Mean (SD) age of the participants was 43.9(11.90) years. Out of 525 participants, majority were females (57.3%, n = 301). Approximately half of the participants (44.2%, n = 232) were of age 31–45 years, and a majority (81.1%, n = 426) had no formal education. Most of the participants (81.3%, n = 427), visited hospitals for diagnosis because of symptomatic appearance of HCV infection (Table 1).

**Table 1:** Sociodemographic and clinical characteristics of the participants

Categories	Frequency n = 525	Percentage (%)
Sex		
Male	224	42.7
Female	301	57.3
Age		
18–30 years	74	14.1
31–45 years	232	44.2
46–60 years	171	32.6
≥ 61 years	48	9.1
Marital status		
Married	510	97.2
Unmarried	11	2.1
Widow(er)	4	0.7
Education		
Illiterate	426	81.1
Primary	45	8.6
Islamic	8	1.5
High School	28	5.3
College	12	2.3
University	6	1.1
Job Status		
Jobless	30	5.7
Stay at Home married female	294	56
Daily Wagers^a^	127	24.2
Self-Employed	61	11.6
Government	13	2.5
Income Per Month (PKR)*		
≤ 10,000	89	16.9
11,000–20,000	405	77.1
21,000–30,000	24	4.6
31,000–40,000	5	1
41,000–50,000	2	0.4
Previous Therapy		
No	328	62.5
Yes	197	37.5
Duration of illness		
≤ 1 year	235	44.8
2–3 years	176	33.5
≥ 4 years	114	21.7
Presence of liver cirrhosis		
No	452	86.1
Yes	73	13.9
Reason for HCV testing		
Precaution	11	2.1
Pre-travel	5	0.9
Pre-blood donation	5	0.9
Pre dental extraction	26	5
Pregnancy	26	5
Pre-Op	25	4.8
Typical Symptoms	427	81.3

^a^PKR = Monthly family income in Pakistani rupees; Pre-Op: Pre-surgical procedure; Daily Wagers: workers who are given money for an 8-h physical work per day on daily basis; Previous therapy: experienced failed interferon-based treatment in the past; Typical symptoms: Symptoms related to HCV infection like fatigue, fever, poor appetite, muscle pain, nausea etc

### Brief illness perception questionnaire (BIPQ) study variables

Results showed a good internal consistency of α = 0.859. The mean (SD) overall sum score of BIPQ was 43.35(13.15). Participants scored highest on the “concern” scale with a mean score of 8.08(2.09) and lowest on “Coherence” scale with a mean score of 2.92(1.86). Participants generally believed in control of HCV infection with DAAs and scored 7.92(2.13) on “treatment control” but also portrayed emotional problems due to HCV infection with a mean score of 6.13(2.27). (Table 2).

**Table 2:** Descriptive statistics of Brief Illness Perception Questionnaire Item scales

Study Item	Mean (SD) (N = 525)
Consequences ^n^	5.07 (2.67)
Timeline ^n^	5.44 (2.41)
Personal Control ^p^	5.50 (2.64)
Treatment Control ^p^	7.92 (2.13)
Identity ^n^	4.97 (2.36)
Concern ^n^	8.08 (2.09)
Coherence ^p^	2.92 (1.86)
Emotional Representation ^n^	6.13 (2.27)
Overall Score ^n^	43.35 (13.15)

SD Standard Deviation; n study items represent negative perception; p study items represent positive perception. Score range of all items is from 0 to 10. Overall score range is from 0 to 80

Females showed higher “emotional representation” with a mean (SD) score of 6.43(2.13) than males, who scored 5.85(2.13) with a significant difference of *p* = 0.04. Similarly, females showed lower “coherence” 2.69(1.59) than males 3.24(2.13), *p* = 0.006. Participants who experienced failed IFN based anti-HCV treatments in the past reflected less trust in “treatment control” of HCV infection, showing a mean (SD) score of 7.62 (2.33), which was comparatively lower than the score of treatment naïve participants, 8.11(1.99), p = 0.029. Similarly, individuals with HCV who had experience of failed IFN based treatments scored higher on the disease “timeline” believing that HCV infection lasts for a long time than treatment naïve participants scoring 5.17(2.35) with *p* ≤ 0.01. Moreover, participants with daily wage jobs showed higher “emotional representation” and “concern”, with mean (SD) scores of 6.14(2.17) and 8.17(2.02) respectively than participants of any other occupation. Individuals with four years or longer estimated duration of their HCV infection scored significantly higher 6.27(2.50), on timeline than individuals with up to one year of duration of illness, 5.36 (2.53), p = 0.001, believing that HCV is a long-term illness. Furthermore, they presented poor perception about treatment control with DAAs and scored significantly lower 7.36(2.30), p ≤ 0.01, than those with shorter duration of HCV infection 7.83(2.25). Individuals having liver cirrhosis scored higher on timeline scale 6.21(2.33) than those with no liver cirrhosis 5.32(2.39), p = 0.004, and their identity score 5.84(2.14) was statistically significant (p = 0.04) than individuals with no liver cirrhosis 4.89(2.38). (Additional file 1: Table S1).

Multivariable linear regression results indicated that no independent variable could significantly determine the plausible cause of higher overall score of the participants (Table 3).

**Table 3:** Regression analysis for BIPQ overall score with sociodemographic and clinical characteristics of participants

Characteristics	Univariable estimates		Multivariable estimates	
	B (95% CI)	p-value	B (95%CI)	p-value
Age (years)				
18–30^R^				
31–45	− 1.29 (− 4.71 to 2.13)	0.460	− 1.12 (− 4.69 to 2.43)	0.534
46–60	2.54 (− 1.02 to 6.11)	0.162	2.31 (− 1.56 to 6.18)	0.242
≥ 61	2.62 (− 2.12 to 7.38)	0.278	1.83 (− 3.43 to 7.08)	0.496
Sex				
Female ^R^				
Male	− 2.30 (− 4.57 to − 0.02)	0.047	− 2.62 (− 7.71 to 2.45)	0.311
Qualification				
Illiterate ^R^				
Primary	− 2.76 (− 6.78 to 1.25)	0.177	− 0.097(− 4.53 to 4.34)	0.966
Islamic	− 1.91 (− 11.07 to 7.23)	0.681	− 1.17 (− 10.39 to 8.05)	0.803
High School	− 7.81 (− 12.81 to − 2.81)	0.002	− 3.71 (− 9.41 to 1.98)	0.201
College	− 2.08 (− 9.59 to 5.42)	0.586	1.28 (− 7.16 to 9.72)	0.766
University	− 7.66 (− 18.21 to 2.87)	0.154	− 8.04 (− 21.55 to 5.46)	0.242
Job status				
Jobless ^R^				
Daily wages	− 0.44 (− 3.11 to 2.22)	0.743	1.65 (− 3.71 to 7.02)	0.544
Self Employed	− 7.31 (− 10.86 to − 3.75)	0.000	− 4.47 (− 10.36 to 1.42)	0.136
Government	− 1.92 (− 9.13 to 5.28)	0.599	3.29 (− 6.86 to 13.45)	0.524
Income per month (PKR)				
≤ 10,000 ^R^				
11–20,000	− 1.46 (− 4.47 to 1.55)	0.343	− 0.60 (− 3.71 to 2.51)	0.704
21–30,000	− 3.32 (− 9.25 to 2.61)	0.271	1.62 (− 4.61 to 7.86)	0.608
31–40,000	− 13.14 (− 24.99 to − 1.28)	0.030	− 8.88 (− 21.02 to 3.26)	0.151
41–50,000	3.25 (− 15.18 to 21.69)	0.729	4.95 (− 14.36 to 24.28)	0.614
Duration of illness				
≤ 1 year ^R^				
2–3 years	− 1.90 (− 4.46 to 0.66)	0.146	− 2.12 (− 4.73 to 0.49)	0.112
≥ 4 years	2.02 (− 0.91 to 4.95)	0.177	1.14 (− 2.49 to 4.78)	0.537
Presence of Liver cirrhosis				
No ^R^				
Yes	3.07 (− 0.17 to 6.32)	0.064	0.34 (− 3.62 to 4.32)	0.863
Previous therapy				
No ^R^				
Yes	2.42 (0.09 to 4.74)	0.041	1.92 (0.56 to 4.41)	0.130

B: Unstandardized coefficient; ^R^ Reference group in the category; p-value: *p* ≤ 0.05; PKR: Pakistani rupees; 95%CI: 95% Confidence interval (lower limit to upper limit)

**Fig. 1:**
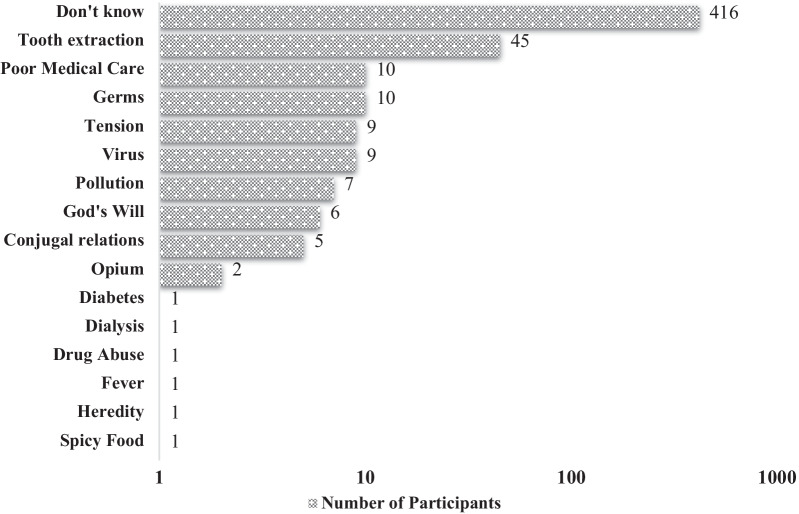
Number of participants and their perception based reported HCV Causality factors (Total Participants = 525)

The ninth item of BIPQ asking about the three most important causality factors that participants thought to have caused their HCV infection was either answered with “don’t know” or only one HCV causality factor. Four hundred and sixteen (n = 416) participants of this study making 79.3% of the studied population, answered “don’t know” when asked about their HCV causality risk factor. Only 8.6% of participants (n = 45) stated that unhygienic dental extraction procedures were the cause of their HCV infection, followed by 2% of participants (n = 10) considering poor medical care as their source of HCV transmission. Other factors in which participants believed to have caused their infection are shown in Fig. 1.

## Discussion

This is the first study to examine the participants’ perception about HCV infection and its implications for treatment in KPK Pakistan. This study explored the impact of participants’ sociodemographic and clinical characteristics on their perception of hepatitis C infection. Most of the participants, who visited government healthcare facilities in KPK Pakistan, had no formal education and more than one-third had a history of HCV treatment failure.

This study showed that participants perceived HCV infection as a deadly illness and were concerned about their future health. This condition was particularly prominent in individuals who went through failed IFN based HCV treatment regimens in the past. They believed in higher chronicity of hepatitis C infection that is, believing that HCV infection lasts for a long time. Such participants also presented more negative perception about the treatment control of HCV infection with DAAs. One of the reasons might be due to lack of symptomatic relief with previous IFN based therapies, as observed in other studies [36]. Individuals with liver cirrhosis were more likely to have experienced more severe HCV-related symptoms and hence were more concerned about disease progression. Similar findings were observed in a study conducted in individuals with HCV in Thailand [37]. Literature shows that knowledge regarding the success of a treatment regimen can motivate individuals with HCV to initiate and complete therapy [38]. Most of HCV treatment regimens require patients to self-administer their doses. Therefore, positive perception to ensure adherence through patient motivation and self-regulation is required to obtain optimum therapeutic outcomes. Langston et al. [10] observed that positive perception about treatment control could help successfully to engage the individuals in self-management of their HCV infection, which led to significant adherence-based coping behaviours. Improving perception has successfully improved patient-reported treatment outcomes in disease conditions like diabetes and AIDS [39]. Therefore, provision of educational counselling by healthcare professionals is likely to improve patient perception, which can assist in achieving a better patient-oriented treatment outcome [40].

The emotional turmoil that HCV brought to the participants was perceived differently by different groups. Female participants showed higher emotional representation than males. This finding is quite different from the notion that being social enables women to deal with their emotional distress better than men [41]. However, the majority of females with HCV infection in this study were stay-at-home wives and their access to information on HCV and its transmission risk factors was likely limited. These findings are in line with the results of a health survey conducted previously in Pakistan [42]. On the contrary, it could be related to HCV related stigma in women, fearing that their marital life might be affected, or they might be discriminated by family or society, and they would have to deal with disappointment and isolation. These observations are supported by a health survey previously conducted in twin cities (Islamabad and Rawalpindi) of Pakistan [43].

The majority of the study cohort was illiterate and presented to hospitals only upon symptomatic appearance of HCV infection. Such participants, particularly those with daily wage jobs, showed a deeper concern for being infected with HCV infection and showed more emotional representation than those of other occupations. Daily wagers seek odd jobs daily, which usually requires physical work. Therefore, deteriorating health due to undiagnosed HCV infection prevented them to perform their tasks effectively. They were worried about losing the only income source (physical health) to support their families economically. This concern for loss of economic support or loss of productivity due to HCV infection as consequence was also observed in Malaysian population [44]. This study highlighted that participants were lacking adequate knowledge about HCV causative and transmission risk factors. Only a small minority of study participants indicated that unsanitary and non-sterile dental practices were responsible for their HCV infection. Whilst such observations are supportive of previously published studies about HCV transmission risk factors in Pakistan [45, 46], having knowledge of such factors and actual behaviour modification to avoid HCV infection can be unrelated. A national survey conducted in Egypt highlighted that despite having basic knowledge related to hepatitis C, rate of HCV infection was high in the Egyptian population [47].

Individuals’ social and cognitive motivation and ability to gain access to, understand, and use disease-oriented information conveniently in ways to promote and maintain good health is referred to as health literacy [48]. Patients' health-seeking behaviour in self-management of chronic diseases is markedly affected by health literacy [49]. Addressing patient perception through educational intervention has been shown to significantly improve their quality of life in chronic disorders [50, 51]. Healthcare professionals including physicians and pharmacists can counsel patients accordingly and can effectively improve patient-oriented outcomes. In a randomized controlled trial in Pakistan, educational intervention led by a pharmacist has successfully improved the quality of life and drug compliance in a major cohort of individuals with HCV [52]. Moreover, educating individuals with HCV infection is associated with positive health outcomes in various HCV care models, including generation of patient interest in treatment, increased disease-specific knowledge, willingness to accept anti-HCV treatments, and increased liver specialty care clinic attendance [53].

Individuals with HCV vary in their demographic and clinical characteristics, so do their illness perceptions and health-seeking behaviour. It is up to the understanding of health care providers to improve illness perception in these individuals to optimize their treatment compliance and treatment outcomes.

### Strengths and limitations of study

It is the first of its kind study to explore the link of HCV perception with treatment outcomes in KPK Pakistan. Hospitals were selected from two districts of KPK, Charsadda and Peshawar, as these hospitals were most frequently visited by the public of each district, hence these sites cover a larger representation of the general population.

However, this study had few limitations. Some of the questionnaire items like emotional representation, consequences, and treatment control needed counselling and follow up of participants to monitor improvement in their perception, self-regulation, and health care outcomes. Second, being diagnosed with HCV made the status of participants change from ‘unaware’ to ‘aware’ which could be a contributing factor to influence their perception. Third, majority of the participants in this study were women and had no formal education. Therefore, the individuals included in this study might not be generalizable to other populations in the community.

## Conclusion

This study showed that most of the participants had negative or threatening beliefs regarding HCV infection. Individuals with liver cirrhosis believed that HCV infection is a long-term illness. Participants who have had a previously failed IFN based treatment presented poor perception about treatment control of DAAs. Females showed relatively greater emotional representation than male participants. Healthcare providers should take these issues into consideration when helping individuals with HCV to increase their adherence to DAA-based treatment and increase their chance of achieving optimal therapeutic outcomes.

## Supplementary Information

**Additional file 1: Table S1.** Comparative analysis of BIPQ items among the sociodemographic and clinical characteristics of study participants.

## Data Availability

The datasets used and/or analysed during the current study are available from the corresponding author on reasonable request.

## Abbreviations

HCV: Hepatitis C virus
RNA: Ribonucleic acid
PCR: Polymerase chain reaction
WHO: World Health Organization
BIPQ: Brief Illness Perception Questionnaire
AIDS: Acquired immune deficiency syndrome
KPK: Khyber Pakhtunkhwa
IFN: Interferon
SVR: Sustained virologic response
