# Supplementary material for: Illness perception about hepatitis C virus infection: a cross-sectional study from Khyber Pakhtunkhwa Pakistan
Source: BMC Infect Dis. 2022 Jan 21;22:74. doi: 10.1186/s12879-022-07055-5 (PMC8783479; doi:10.1186/s12879-022-07055-5)
Supplement: Supplementary file 1 — Additional file 1: Table S1. Comparative analysis of BIPQ items among the sociodemographic and clinical characteristics of study participants. [file 12879_2022_7055_MOESM1_ESM.docx]

Additional Table 1. Comparative analysis of BIPQ items among the sociodemographic and clinical characteristics of study participants.

| **Category** | **Consequences** | **T** | **P** | **Timeline** | **T** | **P** | **Your Control** |  | **P** | **Treatment Control** | **T** |  | **P** | **Identity** | **T** | **P** | **Concern** | **T** | **P** | **Coherence** | **T** | **P** | **Emotional Representation** | **T** | **P** |
| --- | --- | --- | --- | --- | --- | --- | --- | --- | --- | --- | --- | --- | --- | --- | --- | --- | --- | --- | --- | --- | --- | --- | --- | --- | --- |
| **Sex ^a^** | | | | | | | | | | | | | | | | | | | | | | | | | |
| Female | 5.06(2.66) | 34245.5 | 0.75 | 5.59(2.41) | 31295.5 | 0.15 | 5.31(2.58) | 37043.5 | 0.05* | 7.89(2.0) | 35736.5 |  | 0.23 | 4.96(2.2) | 33989.5 | 0.87 | 8.27(1.8) | 31021.5 | 0.11 | 2.69(1.59) | 38206.5 | 0.006** | 6.34(2.13) | 30230.5 | 0.04* |
| Male | 5.09(2.69) |  |  | 5.25(2.39) |  |  | 5.75(2.70) |  |  | 7.97(2.3) |  |  |  | 4.98(2.5) |  |  | 7.83(2.3) |  |  | 3.24(2.13) |  |  | 5.84(2.42) |  |  |
| **Age ^b^** | | | | | | | | | | | | | | | | | | | | | | | | | |
| 18-30 years | 5.12(2.69) | 42.93 | 0.026* | 5.27(2.41) | 10.65 | 0.014* | 5.49(2.58) | 3.39 | 0.33 | 7.91(2.1) | 3.06 |  | 0.38 | 5.05(2.2) | 4.26 | 0.23 | 7.89(1.9) | 12.68 | 0.005** | 3.22(2.11) | 1.36 | 0.71 | 6.12(2.28) | 8.24 | 0.04* |
| 31-45 years | 4.96(2.66) |  |  | 5.11(2.38) |  |  | 5.67(2.62) |  |  | 8.14(1.9) |  |  |  | 4.75(2.3) |  |  | 7.86(2.2) |  |  | 2.88(1.79) |  |  | 5.84(2.36) |  |  |
| 46-60 years | 5.47(2.65) |  |  | 5.82(2.32) |  |  | 5.20(2.72) |  |  | 7.68(2.30) |  |  |  | 5.11(2.3) |  |  | 8.42(2.1) |  |  | 2.84(1.78) |  |  | 6.32(2.10) |  |  |
| 61-75 years | 5.07(2.67) |  |  | 6.00(2.57) |  |  | 5.75(2.50) |  |  | 7.77(2.29) |  |  |  | 5.46(2.3) |  |  | 8.23(1.8) |  |  | 2.96(2.12) |  |  | 6.81(2.18) |  |  |
| **Qualification^b^** | | | | | | | | | | | | | | | | | | | | | | | | | |
| Illiterate | 5.19(2.65) | 6.46 | 0.26 | 5.51(2.73) | 5.35 | 0.37 | 5.33(2.59) | 16.94 | 0.005** | 7.92(2.12) | 9.81 |  | 0.08* | 5.03(2.3) | 3.49 | 0.62 | 8.24(1.97) | 16.06 | 0.007** | 2.81(1.75) | 10.4 | 0.06 | 6.24(2.19) | 8.96 | 0.11 |
| Primary | 5.02(2.61) |  |  | 5.49(2.38) |  |  | 5.89(2.54) |  |  | 7.78(1.96) |  |  |  | 4.89(2.5) |  |  | 7.53(2.59) |  |  | 3.64(2.39) |  |  | 5.78(2.45) |  |  |
| Islamic | 4.13(2.16) |  |  | 5.13(2.35) |  |  | 6.13(2.99) |  |  | 7.00(2.13) |  |  |  | 4.63(1.9) |  |  | 7.38(1.2) |  |  | 2.2(5.46) |  |  | 6.38(1.40) |  |  |
| High School | 4.14(3.10) |  |  | 4.43(2.60) |  |  | 7.36(2.54) |  |  | 8.54(2.32) |  |  |  | 4.71(2.7) |  |  | 7.39(2.6) |  |  | 3.71(2.58) |  |  | 5.29(2.91) |  |  |
| College | 4.50(3.03) |  |  | 5.08(2.87) |  |  | 5.17(3.24) |  |  | 7.50(2.54) |  |  |  | 4.75(3.1) |  |  | 6.75(2.5) |  |  | 2.50(1.00) |  |  | 6.17(2.40) |  |  |
| University | 3.83(1.72) |  |  | 6.17(2.99) |  |  | 6.00(2.45) |  |  | 8.83(1.32) |  |  |  | 3.33(1.8) |  |  | 7.50(1.5) |  |  | 3.50(1.37) |  |  | 4.00(2.09) |  |  |
| **Job Status^b^** | | | | | | | | | | | | | | | | | | | | | | | | | |
| Jobless | 5.27(2.77) | 6.92 | 0.14 | 5.63(2.41) | 5.42 | 0.24 | 5.27(3.17) | 13.33 | 0.01** | 7.20(2.61) | 5.44 |  | 0.24 | 5.13(2.4) | 4.72 | 0.32 | 8.07(2.3) | 9.71 | 0.04* | 3.20(2.20) | 7.19 | 0.13 | 6.53(2.24) | 13.76 | 0.008** |
| Stay at Home Wife | 5.06(2.68) |  |  | 5.59(2.43) |  |  | 5.33(2.56) |  |  | 7.94(1.99) |  |  |  | 4.95(2.2) |  |  | 8.28(1.8) |  |  | 2.68(1.58) |  |  | 6.34(2.13) |  |  |
| Daily Wages | 5.45(2.44) |  |  | 5.43(2.18) |  |  | 5.39(2.57) |  |  | 7.97(2.19) |  |  |  | 5.27(2.4) |  |  | 8.17(2.0) |  |  | 3.16(1.99) |  |  | 6.14(2.17) |  |  |
| Self-Employed | 4.30(2.86) |  |  | 4.70(2.48) |  |  | 6.69(2.54) |  |  | 8.23(2.17) |  |  |  | 4.44(2.6) |  |  | 6.98(2.9) |  |  | 3.39(2.39) |  |  | 4.97(2.71) |  |  |
| Government | 4.77(3.08) |  |  | 5.46(3.33) |  |  | 5.38(3.07) |  |  | 7.38(2.93) |  |  |  | 4.62(3.1) |  |  | 7.85(1.7) |  |  | 3.15(2.07) |  |  | 5.69(2.75) |  |  |
| **Income per month (PKR)**^b^ | | | | | | | | | | | | | | | | | | | | | | | | | |
| < 10,000 | 5.18(2.70) | 4.93 | 0.35 | 5.52(2.59) | 6.80 | 0.14 | 4.94(2.72) | 10.30 | 0.03* | 7.54(2.32) | 5.07 |  | 0.28 | 5.11(2.2) | 5.50 | 0.24 | 7.99(2.18) | 7.01 | 0.13 | 2.79(1.59) | 2.81 | 0.59 | 6.21(2.14) | 5.27 | 0.26 |
| 11,000-20,000 | 5.07(2.71) |  |  | 5.43(2.39) |  |  | 5.56(2.63) |  |  | 7.98(2.08) |  |  |  | 4.97(2.3) |  |  | 8.14(2.1) |  |  | 2.92(1.91) |  |  | 6.13(2.30) |  |  |
| 21,000-30,000 | 5.04(1.94) |  |  | 5.46(1.79) |  |  | 6.71(2.01) |  |  | 8.17(2.37) |  |  |  | 5.08(1.9) |  |  | 7.83(1.7) |  |  | 3.46(2.00) |  |  | 6.33(1.95) |  |  |
| 31,000-40,000 | 3.00(1.41) |  |  | 3.80(1.79) |  |  | 5.60(2.70) |  |  | 9.00(1.00) |  |  |  | 2.40(2.3) |  |  | 5.80(2.9) |  |  | 2.60(0.89) |  |  | 3.80(3.03) |  |  |
| 41,000-50,000 | 7.00(4.24) |  |  | 9.00(1.41) |  |  | 4.00(2.83) |  |  | 8.50(0.71) |  |  |  | 5.00(1.4) |  |  | 8.00(1.4) |  |  | 3.00(1.41) |  |  | 4.50(0.71) |  |  |
| **Previous Therapy ^a^** | | | | | | | | | | | | | | | | | | | | | | | | | |
| No | 5.02(2.68) | 33184.0 | 0.60 | 5.17(2.35) | 38320.0 | 0.000** | 5.67(2.57) | 29160.0 | 0.06 | 8.11(1.99) | 28701.0 |  | 0.02* | 4.85(2.3) | 35004.0 | 0.10 | 8.03(1.9) | 34997.0 | 0.10 | 2.95(1.9) | 32207.0 | 0.95 | 6.09(2.21) | 33715.0 | 0.39 |
| Yes | 5.16(2.67) |  |  | 5.89(2.44) |  |  | 5.22(2.72) |  |  | 7.62(2.32) |  |  |  | 5.18(2.4) |  |  | 8.16(2.2) |  |  | 2.88(1.79) |  |  | 6.20(2.3) |  |  |
| **Presence of liver cirrhosis^a^** | | | | | | | | | | | | | | | | | | | | | | | | | |
| No | 5.03(2.71) | 17538.0 | 0.42 | 5.32(2.39) | 19952.0 | 0.004* | 5.54(2.61) | 15463.0 | 0.38 | 7.93(2.10) | 16787.0 |  | 0.81 | 4.89(2.3) | 18915.0 | 0.04* | 8.01(2.1) | 18994.0 | 0.03* | 2.94(1.84) | 15465.0 | 0.36 | 6.08(2.2) | 17545.0 | 0.37 |
| Yes | 5.32(2.40) |  |  | 6.21(2.33) |  |  | 5.26(2.83) |  |  | 7.86(2.31) |  |  |  | 5.48(2.1) |  |  | 8.52(1.83) |  |  | 2.84(1.98) |  |  | 6.44(2.1) |  |  |
| **Duration of illness^b^** | | | | | | | | | | | | | | | | | | | | | | | | | |
| ≤ 1 year | 5.21(2.69) | 1.66 | 0.43 | 5.36(2.30) | 21.29 | 0.000** | 5.56(2.53) | 0.81 | 0.66 | 7.83(2.25) | 17.44 |  | 0.000** | 4.89(2.2) | 4.35 | 0.11 | 8.13(1.99) | 0.081 | 0.96 | 2.84(1.76) | 3.49 | 0.17 | 6.20(2.1) | 0.60 | 0.74 |
| 2-3 years | 4.86(2.64) |  |  | 5.02(2.35) |  |  | 5.54(2.70) |  |  | 8.41(1.70) |  |  |  | 4.83(2.3) |  |  | 8.03(2.14) | 21.48 |  | 3.13(2.01) |  |  | 5.99(2.3) |  |  |
| ≥ 4 years | 5.12(2.68) |  |  | 6.27(2.50) |  |  | 5.31(2.76) |  |  | 7.36(2.30) |  |  |  | 5.37(2.5) |  |  | 8.05(2.21) |  |  | 2.76(1.81) |  |  | 6.18(2.4) |  |  |
| **Reason for HCV testing^a^** | | | | | | | | | | | | | | | | | | | | | | | | | |
| Precaution | 3.91(2.66) | 5.88 | 0.43 | 4.36(1.91) | 11.12 | 0.08 | 7.00(2.32) | 6.14 | 0.41 | 8.73(1.35) | 8.95 |  | 0.17 | 3.82(1.7) | 5.83 | 0.44 | 6.18(2.60) |  | 0.002** | 2.36(0.81) | 8.83 | 0.18 | 4.73(2.5) | 10.57 | 0.10 |
| Pre-travel | 5.60(1.52) |  |  | 6.20(2.59) |  |  | 6.00(2.55) |  |  | 6.60(3.71) |  |  |  | 5.20(1.9) |  |  | 7.80(0.45) |  |  | 2.00(0.71) |  |  | 5.40(1.6) |  |  |
| Pre-blood donation | 3.20(3.11) |  |  | 6.00(2.55) |  |  | 6.20(3.11) |  |  | 7.00(1.58) |  |  |  | 3.80(3.0) |  |  | 5.40(3.13) |  |  | 4.20(3.27) |  |  | 3.00(3.2) |  |  |
| Pre-dental extraction | 4.50(2.66) |  |  | 5.27(2.75) |  |  | 5.23(2.57) |  |  | 7.88(1.6) |  |  |  | 4.58(2.9) |  |  | 7.69(2.54) |  |  | 2.81(2.29) |  |  | 6.00(2.3) |  |  |
| Pregnancy | 5.42(2.73) |  |  | 6.15(2.52) |  |  | 5.27(2.58) |  |  | 7.27(2.3) |  |  |  | 5.12(2.4) |  |  | 8.04(1.59) |  |  | 2.96(2.44) |  |  | 6.42(2.2) |  |  |
| Pre-Op | 5.24(2.95) |  |  | 6.44(2.26) |  |  | 6.20(2.38) |  |  | 7.60(2.2) |  |  |  | 4.92(2.4) |  |  | 7.24(2.74) |  |  | 3.28(2.05) |  |  | 6.20(2.5) |  |  |
| Typical Symptoms | 5.12(2.66) |  |  | 5.37(2.38) |  |  | 5.44(2.66) |  |  | 7.99(2.1) |  |  |  | 5.03(2.3) |  |  | 8.24(1.98) |  |  | 2.92(1.78) |  |  | 6.19(2.2) |  |  |

*PKR Pakistani rupee; ^a^ Mann-Whitney U test; ^b^ Kruskal-Wallis test; Duration of illness: Time after HCV RNA PCR test is positive; T= Test statistic; *significance at p ≤ 0.05; ** significance at p ≤ 0.01.
